# High frequency of deficient consumption and low blood levels of 25-hydroxyvitamin D in HIV-1-infected adults from São Paulo city, Brazil

**DOI:** 10.1038/srep12990

**Published:** 2015-08-10

**Authors:** Stephanie Hael Sales, Sandra da Matta, Daniela Cardeal da Silva, Tatiane Assone Assone, Luiz Augusto M. Fonseca, Alberto J. S. Duarte, Jorge Casseb

**Affiliations:** 1 Laboratory of Dermatology and Immunedeficiencies, Department of Dermatology, University of São Paulo Medical School, Brazil; 2 Institute of Tropical Medicine, São Paulo University, São Paulo, Brazil; 3 Department of Preventive Medicine – São Paulo University Medical School.

## Abstract

Micronutrient deficiency is common in patients with HIV/AIDS, usually caused by mal-absorption and/or drug interactions. 25-hydroxyvitamin D is of fundamental importance for the homeostasis of musculoskeletal health. The current study aimed to evaluate the nutritional status of HIV-infected subjects in order to make their nutritional diagnoses, including their vitamin D blood levels, and to estimate their consumption of vitamin D. The study included 98 HIV-1-infected subjects, followed at University of São Paulo Medical School - HC-FMUSP. We performed a nutritional evaluation, along with the determination of patients’ serum 25-hydroxyvitamin D and calcium concentration, biochemical analyses, and an anthropometric assessment. In the medical interview a 24-hour food recall was used (R24) to estimate daily calorie intake, macronutrients, calcium, and vitamin D. A high level of vitamin D deficiency was observed in our patients: 83.4% of them had levels below 30 ng/ml; they also presented an increased risk of cardiovascular disease, along with a high consumption of dietary fat. Factors related to the virus itself and to the use of antiretroviral drugs may have contributed for the low vitamin D levels seen in our HIV-1-infected patients.

HIV-infected patients on highly active antiretroviral therapy (HAART) have an increased risk for several complications not directly related to AIDS, many of them more common in aging patients^1^,^2^, such as cardiovascular disease, cancer, kidney and bone disease^3^. There is a decrease in bone mineralization in a large proportion of patients, resulting from various factors from the host itself, the virus and the use of HAART. Appropriate nutritional status is a prerequisite for improving the quality of life of these patients^4^.

Micronutrient deficiency is common in HIV/AIDS patients, caused by mal-absorption, drug interactions, metabolic changes and loss of fluids from vomiting and diarrhea. Vitamins and minerals are considered essential to maintaining health, as they protect against opportunistic infections, and favor the body’s proper functioning, particularly that of the immune system^5^. Vitamin D is of fundamental importance for the homeostasis of calcium and phosphorus, and for musculoskeletal health^6^,^7^,^8^. Furthermore, vitamin D deficiency causes an increase in parathyroid hormone (PTH), which in turn increases insulin resistance, leading to hypertension, inflammation and increased cardiovascular risk^9^.

The usual daily intake of vitamin D varies between 5–10 mg; it can be found in foods such as fish oil, egg yolk and milk^10^. An adequate consumption of calcium and vitamin D from food and/or supplements is necessary to achieve a normal bone mineral density (BMD), in order to decrease the rate of bone loss in the elderly^10^. The current study aimed to evaluate the nutritional status of HIV-infected subjects followed in a Sao Paulo, Brazil university hospital, in order to make their nutritional diagnoses, including their vitamin D blood levels, and to estimate their vitamin D consumption.

## Material and Methods

We have been following HIV-positive patients in our outpatient service for 29 years. For the purpose of this study, from a total of 500 HIV-1 subjects we currently follow, a subset of 98 were invited and accepted to participate. In this convenience sample, patients were included if they were more than 18 years old and were in active follow-up during the period ranging from August 2011 to December 2013. After signing an informed consent form, patients answered a questionnaire containing information on their socio-behavioral characteristics.

A detailed nutritional evaluation was performed, along with the determination of 25-hydroxyvitamin D, calcium, cholesterol and its fractions, triglycerides and glucose blood levels, in order to make a nutritional diagnosis and to estimate patients consumption of vitamin D. Vitamin D levels were classified according to the American Society of Endocrinology as: <10 ng/ml – deficiency; 10–30 ng/ml – insufficiency; and >30 ng/ml – sufficiency. Sun exposure was considered adequate if legs and/or arms were exposed to sunlight for at least 20 minutes every day.

Anthropometric assessment consisted of measuring the triceps, biceps, sub-scapular, axillary, calf average and mid-thigh skin folds, the arm and waist circumferences, and body weight and height^11^. In the medical interview a 24-hour food recall was used (R24), in order to estimate the daily caloric intake, macronutrients, calcium and vitamin D. Questionnaires also included questions about cardiovascular risk factors, such as smoking.

Descriptive statistics were performed with the statistics program SPSS 20.0 and displayed in tables showing frequencies and percentages. Statistical analysis was conducted using Student’s t-test for parametric data, and the chi-square test for proportions. Possible differences in patient characteristics or laboratory values among groups were evaluated with two-way Mann-Whitney’s test and Kruskal-Wallis test. The study was approved by the ethical board of Hospital das Clínicas, FMUSP (CAPPesq no 0532/11), and all procedures were done in accordance with approved guidelines.

## Results

Mean age was 39 years for the 66 men (67.4%) and 41.7 years for the 32 (32.6%) women. Mean duration of HIV infection was 6.31 years for men and 7.31 for women; smokers represented 22.6% of the sample and most were on HAART (Table 1). Table 1 also shows the blood levels of 25-hydroxyvitamin D and ionic calcium: 21 (66%) of women and 49 (72%) of men presented deficient levels of vitamin D. Similar results were obtained for the consumption of vitamin D, since 26 (81%) of women and 57 (87%) of men did not consume enough daily amounts of foods rich in vitamin D. Ionic calcium levels were normal for 23 (72%) women and 48 (73%) men, respectively.

Table 2 shows the risk factors associated with vitamin D deficiency, classified by gender. Fourteen (43.7%) and 34 (51.5%) of women and men, respectively, reported a minimum sun exposure of 15 minutes on their arms and legs daily. Twenty-six (81.2%) women and 45 (68.2%) men were former or current smokers. One third of the men did consume alcohol three times a week, although no further detailed intake information was obtained. Nine (9.4%) and 18 (27.3%) women and men, respectively, were not on HAART, and 70% of the patients had undetectable viral loads in their blood.

Table 3 presents a nutritional and self-image evaluation of the patients; 20 (63%) women and 33 (50%) men were overweight or obese (p = 0.04). Abdominal circumference, as a measure of cardiovascular risk, was elevated for 23 (72%) women and 29 (53%) men. A self-image evaluation revealed that 16 women and almost 70% of the men did not notice any differences in their appearances. No significant differences were observed for the variables age over 50 years-old, T CD4 cells count, level of HIV viremia, cardiovascular risk, smoking habits, alcohol consumption and BMI; the exception was daily sun exposure (p = 0.009) (Table 4).

## Discussion

A high level of 25-hydroxyvitamin D deficiency and insufficiency was observed in our patients since 83.4% of them had levels below 30 ng/ml. A recent report showed a 23% prevalence of vitamin D deficiency in HIV Brazilian patients^12^. In the United States, a study revealed vitamin D deficiency in 74.4% of HIV-positive adults^10^. Taken together, these studies are in accordance with recent epidemiological studies, which pointed to a worldwide occurrence of vitamin D deficiency^7^,^13^,^14^.

In addition to the findings described above other factors related to HIV itself and to the use of antiretroviral drugs can be considered as contributing causes for low vitamin D blood levels^15^,^16^,^17^. We found a low intake of vitamin D, maybe because its natural occurrence in food is small and supplementation of food with this vitamin is not done for the general population^18^,^19^. Vitamin D supplementation with 600–800 UL/day was not enough to normalize the levels of the vitamin in African American and Latin American HIV-infected patients, and to achieve that goal an intake of 1000 to 2000 UL/day would have been required. Studies involving a larger number of subjects are needed to assess the impact of vitamin D supplementation on the co-morbidities of adults with HIV infection^20^.

The major finding of this study was the high frequency (>80%) of insufficient levels of vitamin D, as well as a high consumption of dietary fat, along with a sizable proportion of overweight and obese patients^21^. Thus, nutritional intervention is necessary, possibly including supplementation of vitamin D. There are several causes for the high prevalence of vitamin D insufficiency, even in low-latitude regions. The synthesis of vitamin D is proportional to the area exposed to sunlight and is influenced by environmental factors such as latitude, season, time of day, amount of clouds or the ozone layer, and factors related to the individuals and their habits^22^,^23^. Persons with more melanin in their skin synthesize less pro-vitamin D for the same dose of UV-B. Sunscreen use is another factor impairing the ability to synthesize vitamin D, a correct use of sunscreen with SPF 8 or 15 reducing the synthesis of vitamin D from 95% to 99.9%, respectively^7^.

More than 98% of our patients did not consume the optimum amount of calcium. A study in the Southern Brazil city of Porto Alegre demonstrated that 95% of children and adolescents did not consume the recommended daily amount of calcium, revealing a significant deficit in their calcium supply^24^. The data from our study in São Paulo confirm that people living with HIV/AIDS have a low calcium intake. The importance of calcium in bone mineralization is worth emphasizing, and therefore the low intake of calcium seen in our patients may put them at high risk for fractures. Corroborating this finding, a 60% prevalence of osteopenia or osteoporosis was seen in the original cohort from where our sample was drawn and also observed elsewhere^25^.

This study has potential confounders since some differences in the distribution of independent variables we found may not be specific to HIV-infected patients, but merely reflect what happens in the population at large.

For example, women’s lower prevalence of smoking, a finding of our study, could be only mirroring the same gender difference in the prevalence of smoking in the population of Sao Paulo city.

Concluding, a high level of vitamin D deficiency or insufficiency was observed in 83.4% of our patients, along with high consumption of dietary fat; the prevalence of overweight was high. Thus, these patients may be at high risk for cardiovascular diseases and bone fractures in the future. Therefore, a multidisciplinary team should care for these subjects aiming at prevention and early treatment of these risk factors.

## Additional Information

**How to cite this article**: Sales, S. H. *et al.* High frequency of deficient consumption and low blood levels of 25-hydroxyvitamin D in HIV-1-infected adults from São Paulo city, Brazil. *Sci. Rep.* **5**, 12990; doi: 10.1038/srep12990 (2015).

## Figures and Tables

**Table 1:** Distribution of consumption and absorption of vitamin D and calcium among HIV-infected patients according to gender.

	Females (n.)	(%)	Males (n.)	(%)	P value
Vitamin D levels					
Deficient (<10 ng/dL)	5	15.63	8	12.12	0.7
Insufficient (11–29 ng/dL)	21	65.63	49	71.43	
Sufficient (>30 ng/dL)	6	18.75	9	15.31	
Consumption of vitamin D					
Adequate	6	18.75	9	13.64	0.51
Inadequate	26	81.25	57	86.36	
Calcium levels					
Deficient (<4.6 mg)	8	25	14	21.21	0.8
Suficient (4.6–5.3 mg)	23	71.88	48	72.73	
Over (>5.30 mg)	1	3.13	4	6.06	
Calcium consumption					
Adequate (>1000 mg)	2	6.25	1	1.52	0.2
Inadequate (<1000 mg)	30	93.75	65	98.5	

**Table 2:** Risk factors influencing absorption of vitamin D and calcium among 98 HIV-infected subjects, by gender.

	Females (n.)	(%)	Males (n.)	(%)	P value
Sun exposure					
Yes	14	43.75	34	51.52	0.471
No	18	56.25	32	48.48	
Smoking					
Yes	6	18.75	21	31.82	0.174
No	26	81.25	45	68.18	
Alcohol consumption					
Yes	2	6.25	22	33.3	0.003
No	30	93.75	44	66.7	
HAART					
Yes	29	90.63	48	72.73	0.043
No	3	9.38	18	27.27	
T CD4 cells count					
≤350 cells/ul	6	6.12	8	8.16	0.322
>350 cells/ul	26	26.53	58	59.18	
HIV plasma viral load					
<50 copies/mL	24	75	44	66.67	0.401
>50 copies/mL	8	25	22	33.33	

**Table 3:** Nutritional status and bodily self-image evaluation among 98 HIV-infected subjects.

	Females (n.)	(%)	Males (n.)	(%)	P value
BMI					
Low	1	3.12	**	**	0.04
Normal	11	34.4	33	50	
Overweight	11	34.4	25	37.9	
Obese	9	28.1	8	12.1	
Cardiovascular risk					
Low	3	9.4	13	19.7	0.004
Moderate	6	18.7	24	36.4	
High	23	71.9	29	53	
Abdominal circumference (WHO classification)					
Low	8	25	37	56.06	0.002
Moderate	6	18.7	15	22.7	
High	18	56.2	14	21.2	
Lipodystrophy					
Yes	15	46.9	22	33.3	0.195
No	17	53.1	44	66.7	

**Table 4:** Vitamin D blood levels according to demographic, laboratory, clinical and sun exposure variables.

Vitamin D					
	Insuficient		Normal		P value
Age (years)					
≤50	64	84.21	12	15.8	0.06
>50	19	86.4	3	13.6	
T CD4 cells count					
≤350	12	85.7	2	14.3	0.9
>350	71	84.5	13	15.5	
HIV plasma viral load					
<50 copies/mL	59	86.8	9	13.2	0.4
Detectable	24	80	6	20	
Use of HAART					
No	17	81	4	19	0.6
Yes	66	86	11	15	
Cardiovascular risk					
Low	14	87.5	2	12.5	0.6
Moderate	27	90	3	10	
High	16	76	5	23.4	
Very high	26	84	5	16	
BMI					
Low	1	1.2	0	**	0.4
Normal	35	42	9	60	
Overweight	31	37	5	33	
Obese	16	19	1	7	
Smoking habits					
No smoker	59	83	12	17	0.5
Smoker	24	89	3	11	
Alcohol					
Yes	19	79	5	20.8	0.4
No	64	86.5	10	13.5	
Sun exposure					
Yes	36	75	12	25	0.009
No	47	94	3	6	
