# Erratum: High frequency of deficient consumption and low blood levels of 25-hydroxyvitamin D in HIV-1-infected adults from São Paulo city, Brazil

**DOI:** 10.1038/srep14587

**Published:** 2015-10-20

**Authors:** Stephanie Hael Sales, Sandra Maria Matta, Daniela Cardeal da Silva, Tatiane Assone, Luiz Augusto M. Fonseca, Alberto J. S. Duarte, Jorge Casseb

Scientific Reports 5: Article number: 12990; 10.1038/srep12990 published online: 07102015; updated: 10202015.

The original version of this Article contained a typographical error in the spelling of the author Sandra Maria Matta which was incorrectly given as Sandra da Matta. This has now been corrected in both the PDF and HTML versions of the Article.

In addition, there was a typographical error in the spelling of the author Tatiane Assone which was incorrectly given as Tatiane Assone Assone in the HTML version of this Article. This has now been corrected.

